# Surface treatments of fiber-reinforced posts on the adhesion of resin-based luting agent: An *in vitro* study

**DOI:** 10.6026/973206300210390

**Published:** 2025-03-31

**Authors:** Priyanka Puri, Sakshi Sakshi, Upasana Chhabra, Vaishali Malhotra, Pankaj Bajaj, Yogesh Garg

**Affiliations:** 1 Department of Prosthodontics and Crown and Bridge, PDM Dental College and Research Institute, Bahadurgarh, Haryana, India; 2 Department of Prosthodontics, Geetanjali Dental College and Research Institute, Manav Kheda, Eklingpura, Udaipur, Rajasthan, India; 3 Department of Public Health Dentistry, JCD Dental College, Sirsa, Haryana, India

**Keywords:** Fiber-reinforced posts, resin-based luting agent, bond strength

## Abstract

It is not always true that treating the surface of fiber-reinforced posts will make them bond better. This is especially true where the post bonds with the resin-based luting agent, which is not as strong as where the dentin bonds with the cement. This study was done in a lab to see how different treatments on the post surface affected the bond between a luting agent and a fiber post. For the push-out bond strength test, 50 samples of central incisors were used, with 10 in each group. The samples were further divided into subgroups called cervical, middle and apical third. It was found that the bond strength is increased by chemically treating the fiber posts which increased the mechanical interlocking of luting cement with post surface.

## Background:

Depending on how much tooth structure is still there and how well the restoration process replaces it; the repair can last for a long time. Even though there are many kinds of posts, fiber-reinforced composite (FRC) posts that are prefabricated are becoming more popular [1]. When endodontically treated teeth are restored, fiber posts are used more often than standard cast posts. These posts are made of fiber-reinforced composite (FRC). This is because fiber posts are better than cast posts and cores in many ways, such as being easier to fix quickly and having better biocompatibility, esthetics and resistance to corrosion. It has also been said that root fractures that can't be fixed are less likely to happen with glass fiber posts than with standard metal cast posts [2]. More than one clinical study has shown that post debonding is the most common way that fiber post retained restorations fail. For passive retention of fiber post, resin-based luting agents are the best choice. An etch-and-rinse or self-etch adhesive, or one of the new self-adhesive resin cements, is often used with resin-based luting agents to hold fiber posts in place. It is very important that the luting cement bonds well to the root canal dentin so that the repair works well in the long run [3-4]. Mechanical and chemical methods when used together can change the post-surface by sanding off a layer of epoxy glue with air. The resin mixture has another place to hold on to micromechanically in the spaces between these threads. But several tests showed that bond strength was not better after sandblasting and then silane than after sandblasting alone [5]. A lot of research, though, has shown that the bond is much better in the upper third of the post space dentin [6, 7]. They have shown, on the other hand, that the root canal area does not change how well the post bonds to the canal dentin [8, 9]. So, the point of this lab experiment was to look into how different surface processes of fiber-reinforced composite posts changed the bond strength where the post bonds with the cement.

## Methods and Materials:

This in-vitro study was carried out in the Department of Prosthodontics and Crown & Bridge at PDM Dental College and Research Institute and the push-out bond strength test were done at Spectro-Analytical Lab Ltd, New Delhi. Fifty central incisors were divided into five groups in the study: the control group, 10% hydrogen peroxide, silanization, airborne particle abrasion and airborne particle abrasion followed by silanization. The above groups were further divided into three smaller groups: the cervical third, the middle third and the apical third. The study only included maxillary central incisors that were extracted for periodontal reasons, had straight root canals and had non-carious, fully formed apices. Teeth that were fractured, decayed, or had any calcifications or obstructions were not included. The teeth were cleaned off soft tissue and calculus with an ultrasonic scaler. The teeth were then kept in a 0.5% Chloramine T solution at 4°C for no more than three months. The teeth were washed under running water, dried with a paper towel and put in standard saline at 37°C until they were tested. Protaper rotary nickel-titanium instruments were used for endodontic treatment, which was done using a standard crown- down method. After each change in file size, irrigation was done using 3% NaOCl and 10% EDTA solutions, one after the other. After 24 hours, 240-grit Silicone Carbide (SiC) paper was used to sand down the temporary seal while the area was cooled with water. The coronal gutta-percha was then removed with a pre- shaping drill, leaving a 5-mm long apical seal. Then, a size-3 drill that had already been set up was used to make a 9-mm deep post hole that fit the Rely X fiber post No.3 (3M ESPE, MN, USA). Before being put in, each post was cleaned for 60 seconds with ethanol (99.9 vol %) and then dried completely in the air. Fifty samples were randomly split into five groups of ten each, with names like A, B, C, D and E. For Group A, the post surfaces were not treated in any way. People in Group B put their posts in 10% H2O2 at room temperature for 20 minutes. They were then washed with water and left to dry in the air. For Group C, the post sides were coated with a silane coupling agent (Rely X Ceramic Primer, 3M ESPE, MN, USA) using an applicator tip for 60 seconds.

The coating was spread out in a single layer and it was left to dry. For Group D, 50µm aluminum oxide was sprayed on the post sides for 5 seconds at 2.8 bars. This is called sandblasting. The tip of the sandblasting tool was held 1 cm away from the post and straight up during the process. As part of the process, the post as with Group D, 50µm aluminum oxide was sandblasted onto the post surfaces of Group E. A silane binding agent was then applied in a single layer and left on for 60 seconds. The post surfaces were then dried. With a diamond saw that was cool in water, the part of the root that had the fiber post was cut into two-millimeter-thick pieces at the cervical, middle and apical ends of the root. The circular plunger of the testing machine was used to push each upside-down, cut-off fiber post away from the root dentin in a direction from the crown to the tip. The Universal Testing Machine (Instron, UK) was used to apply a load of 0.5 mm/min with a circular plunger that was 1 mm in diameter to the middle of the post until failure (debonding) happened. After the push-out bond strength test, the samples were looked at under a stereomicroscope at a 40X magnification to find out how the failure happened (debonding). There were three types of failure: cohesive (within the cement), adhesive (between the post and the cement or at the cement/intra-radicular dentin level) and mixed (adhesive and cohesive cracks happened at the same time). We used the statistical package SPSS version 2022 to get frequency tables and measures of central tendency to compare the experimental and control groups across a number of factors at each time point. We used one-way ANOVA (analysis of variance) to compare the mean values of different groups and sub-groups for push-out bond strength measures.

## Results:

Fifty samples were evaluated, with ten in each group. When it came to push-out bond strength, Group E had the most (10.94) and Group A had the least (7.22) (Figure 1 - see PDF). Most push-out bond strength (mean) was seen in Group E, which was made when the post surface was handled with air abrasion and then silanization. After different surface treatments, there was a significant difference (p<0.001) in the mean push-out bond strength of the post. When it came to push-out bond strength, Subgroup 2 had the most (13.75) and Subgroup 1 had the least (1.32). The Subgroup 3 push-out bond strength (mean) was highest, which was found at the root's very tip. There is a significant difference (p<0.001) in the average push-out bond strengths of posts at the cervical, middle and apical parts of the root. In (Table 1), you can see how the push-out link strengths of all three subgroups 1, 2 and 3 compare. All of the samples mostly had problems with the adhesion at the post-cement junction and dentine-cement interface.

## Discussion:

When teeth have been treated with endodontics and a lot of the coronal tooth structure is lost, a post is often placed in the root canal to keep the core for the final restoration. The right restoration for these teeth depends on how strong it is and esthetic it is. Depending on the patient's health, a metal or an esthetic post and core fix may be picked [10- 11]. In the restoration of teeth that have been treated endodontically, fiber posts are being used more and more instead of regular cast posts. They are better than cast posts and cores in many ways, such as being easier to remove and better at biocompatibility, esthetics and resistance to corrosion. They can also be treated more quickly [12]. Compared to traditional metal cast posts, glass fiber posts have also been shown to lower the chance of root fractures that cannot be fixed. This is because their elastic properties are more like those of dentin. This means that stress can be distributed more widely between the tooth and the tissues around it, which keeps the root from fracture [13- 14]. A lot of things can change the contact between the cement and the post, like the type of post, the composite cement and how the post surface was treated before it was used. It is hard to make rules for clinical practices because of this [15]. There is the idea that chemical binding could make the bond stronger. Silane coupling agents are a mix of organic and inorganic molecules that control how well organic and inorganic matrices stick to each other by reacting in two different ways. To improve bonding, it may be suggested to treat the surface with a silane coupling agent before application. Different tests, though, have found different things. Our study found that the mean push-out bond strength was lower on posts that had been treated with air abrasion (Group D) than on posts that had been treated with 10% hydrogen peroxide (Group B). It was statistically important that this difference existed (p<0.05). The findings are similar to those of a study by Khamverdi *et al.* (2011) [16], which looked at how strong the microtensile bond was between a composite core and a fiber post that had been treated on the outside. Ruttonji *et al.* (2019) [17] discovered a statistically significant increase (p < 0.0001) in the bond strength of both fibre and metal posts to resin cement following airborne-particle abrasion with Al2O3 particles and subsequent primer application. Besides that, Kulunk *et al.* (2012) [18] discovered that chemical surface pre-treatment methods were not as good at bond strength as mechanical methods. It's possible that the air abrasion group's stronger bonds are because air abrasion can change the post surface by taking off the resin matrix from that surface. The surface gets rougher and you can see more of the glass strands. The mean push-out bond strength was lower after treating the surface with a silane binding agent (Group C) than after treating the surface with air abrasion (Group D). It was statistically important that this difference existed (p<0.05). In the past, Choi *et al.* (2010) [5] and Gencoglu *et al.* (2013) [19] found the same thing. Stereo microscope with 40X zoom was used to analyze the failure modes.

## Caveats and Limitations:

## There were three types of failures:

[1] Adhesive between the post and cement (no resin cement visible around the post) or between resin cement and root dentin (post encased in resin cement);

[2] Cohesive within the resin cement or post itself

[3] Mixed (adhesive failure at the post-cement/dentin-cement interface and cohesive failure within the cement at the same time) [20-21].

## Conclusion:

The bond strength is improved by both mechanical interlocking and chemical reactions between the luting cements and the post surface, as well as the fiber post moving against it.

## Figures and Tables

**Table 1:** One-way ANOVA results for push out bond strength of all groups and subgroup

**SUBGROUPS**	**GROUPS**	**N**	**Mean**	**Std. Deviation**	**f-value**	**p-value**
Subgroup 1 (Cervical)	GROUP A1	10	3.946	1.92468	33.73	0
	GROUP B1	10	4.379	1.63508		
	GROUP C1	10	5.637	1.24528		
	GROUP D1	10	6.414	1.37276		
	GROUP E1	10	5.334	1.83223		
Subgroup 2 (middle)	GROUP A2	10	7.872	1.60753	78.288	0
	GROUP B2	10	8.031	0.71186		
	GROUP C2	10	8.08	1.04729		
	GROUP D2	10	12.467	0.98109		
	GROUP E2	10	12.518	0.82339		
Subgroup 3 (apical)	GROUP A3	10	9.943	0.60672	30.844	0
	GROUP B3	10	9.278	0.96519		
	GROUP C3	10	10.458	0.9645		
	GROUP D3	10	11.824	0.76462		
	GROUP E3	10	11.982	0.7022		
